# Screening and computational analysis of colorectal associated non-synonymous polymorphism in *CTNNB1* gene in Pakistani population

**DOI:** 10.1186/s12881-019-0911-y

**Published:** 2019-11-07

**Authors:** Suhail Razak, Nousheen Bibi, Javid Ahmad Dar, Tayyaba Afsar, Ali Almajwal, Zahida Parveen, Sarwat Jahan

**Affiliations:** 1 Reproductive physiology lab, Department of Animal Sciences Quaid-i-Azam University, Islamabad, Pakistan; 2 Department of Community Health Sciences, College of Applied Medical Sciences, King Saud University, Riyadh, Saudi Arabia; 3 Department of Bioinformatics, Shaheed Benazir Bhutto Women University, Peshawar, Khyber Pakhtunkhwa Pakistan; 4 College of Science, King Saud University, Riyadh, Saudi Arabia; 5 Faculty of Biological Sciences Quaid-I-Azam University Islamabad and University of Iceland, 101 Reykjavik, Iceland

**Keywords:** CTNNB1, Colorectal cancer, Immunohistochemistry, DNA, Molecular modeling, And protein expression

## Abstract

**Background:**

Colorectal cancer (CRC) is categorized by alteration of vital pathways such as β-catenin *(CTNNB1*) mutations, *WNT* signaling activation*,* tumor protein 53 (*TP53*) inactivation, *BRAF*, Adenomatous polyposis coli (*APC*) inactivation, *KRAS*, dysregulation of epithelial to mesenchymal transition (*EMT*) genes, *MYC* amplification, etc. In the present study an attempt was made to screen *CTNNB1* gene in colorectal cancer samples from Pakistani population and investigated the association of *CTNNB1* gene mutations in the development of colorectal cancer.

**Methods:**

200 colorectal tumors approximately of male and female patients with sporadic or familial colorectal tumors and normal tissues were included. DNA was extracted and amplified through polymerase chain reaction (PCR) and subjected to exome sequence analysis. Immunohistochemistry was done to study protein expression. Molecular dynamic (MD) simulations of CTNNB1^WT^ and mutant S33F and T41A were performed to evaluate the stability, folding, conformational changes and dynamic behaviors of CTNNB1 protein.

**Results:**

Sequence analysis revealed two activating mutations (S33F and T41A) in exon 3 of *CTNNB1* gene involving the transition of C.T and A.G at amino acid position 33 and 41 respectively (p.C33T and p.A41G). Immuno-histochemical staining showed the accumulation of β-catenin protein both in cytoplasm as well as in the nuclei of cancer cells when compared with normal tissue. Further molecular modeling, docking and simulation approaches revealed significant conformational changes in the N-terminus region of normal to mutant *CTNNB1* gene critical for binding with Glycogen synthase kinase 3-B (GSK3) and transducin containing protein1 (TrCp1).

**Conclusion:**

Present study on Pakistani population revealed an association of two non-synonymous polymorphisms in the *CTNNB1* gene with colorectal cancer. These genetic variants led to the accumulation of the *CTNNB1*, a hallmark of tumor development. Also, analysis of structure to function alterations in *CTNNB1* gene is crucial in understanding downstream biological events.

## Background

The worldwide prevalence of colorectal cancer (CRC) is third among cancer incidences in males and fourth among females [1, 2]. In Europe, Colorectal cancer (CRC) is the second most frequent cancer and second foremost source of cancer death after lung cancer, with an estimated overall incidence of 447 per 100,000 [3, 4]. The data obtained from GLOBOCAN 2018, which was produced by the IARC demonstrated that CRC is the third most prevalent malignancy after breast and lung cancer [5]. In 2012 The World Health Organization (WHO) quantified that the age-standardized death rate from CRC was 5.2% in Pakistan. The frequency of CRC is increasing among the native population of Pakistan as observed by a three-fold growth in frequency in males from 2.3 to 6.8% within around 4 years and analogous tendency in females was seen with an increase from 2.5 to 6.7% for the same period [6, 7]. Through recognized scrutiny programs and ensuing initial detection and surgeries of pre-cancerous colonic polyps, the frequency of CRC and its allied deaths have declined over the past 17 years in the countries with the high CRC rate of occurrence [8, 9]. In contrast, the rate of occurrence of CRC in Pakistan showed an increase due to the lack of surveillance programs and insufficient molecular investigations, suggests a theoretically alarming situation developing in the coming decades [10].

CRC arises due to the steady accumulation of modifications in oncogenes and tumor suppressor genes. The accumulation of alterations usually occurs due to the aggregate effects of multiple genetic mutations and epigenetic changes involving genes that are responsible for cell growth and differentiation [11]. These genetic and epigenetic alterations involve different pathways that control multiple biological processes vital to cancer progression [12]. Tumorigenesis of CRC usually results due to differentiation and deregulation of the Wnt/b-catenin signaling pathway which is critical for cell proliferation and migration [13, 14]. Reports revealed that initiating event for the majority of colorectal tumors are the mutations in components of the Wnt/ β-catenin pathway. The activation of the Wnt/β-catenin pathway results in the creation of a free, signaling puddle of β-catenin forms a complex with members of the T-cell factor/lymphoid enhancer-binding factor (TCF/LEF) after entering into the nucleus, initiating transcription of target genes [15]. β-catenin is the main player in cell-cell adhesion by bridging the cytoplasmic tail of cadherins to β-catenin and the actin cytoskeleton. Besides that, β-catenin is a crucial downstream effector in the Wnt/Wingless signaling pathway that governs progression processes such as cell fate specification, proliferation, polarity, and migration [16]. Majority of tumors in colon cancer comprehend defects in the Adenomatous polyposis coli (*APC)* gene that results in β-catenin up-regulation and constitutive signaling by the β-catenin- TCF complex [15]. In tumors deprived of these *APC* mutations [17], the increased levels of β-catenin due to mutations in the NH2 terminus of β- catenin hampers GSK-3β phosphorylation and ensuing degradation by ubiquitin-reliant proteolysis and results in activation of missense mutations at one of the phosphorylation sites at codons S33, S37, S45, and T41 of exon 3 of the *CTNNB1* gene (encoding the β-catenin protein), creating mutant β-catenin that flee phosphorylation and degradation [18]. These amino acids are putative glycogen synthase kinase 3-B (GSK-3β) phosphorylation sites as well as a part of a 6-amino acid stretch, important for ubiquitination, similar to I-kB [19, 20].

The destruction complex is possibly an active multiprotein complex, but its crucial constituents contain, besides to β-catenin itself, the Ser/Thr kinases glycogen synthase kinase 3 (GSK-3) and casein kinase 1 (CK1), the scaffolding protein Axin, the adenomatous polyposis coli (APC) protein, and the E3-ubiquitin ligase β-TrCP. Protein phosphatase 2A (PP2A) also allied with the complex [21]. Mutations in the destruction complex constituents allied with an assortment of cancers result in inappropriate stabilization of β-catenin and Wnt target gene expression in the deficiency of a Wnt stimulus [22]. Mutation in these amino acid residues in exon 3 of the *CTNNB1* gene can create an alleviated form of β-catenin that is not further phosphorylated and degraded and eventually form constitutively active transactivation complexes, which execute to contribute to the loss of cell growth regulation [23, 24]. The exon 3 of *CTNNB1* gene has been screened in different types of tumors and mutations are found in these four residues (for an overview see www.ana.ed.ac.uk/rnusse/pathways/bcatmut.html). These investigations exposed that a mutation in only one of these phosphorylation sites are enough to form a leading constructive form of β-catenin [25–29].

Until now, there was no comprehensive association study of *CTNNB1* gene with colorectal cancer patients in Pakistani Population. In the present study, we screened a *CTNNB1* gene in colorectal cancer samples and investigated the association of *CTNNB1* gene mutations in the development of colorectal cancer. To the best of our knowledge, until now there has been no such study on record for the *CTNNB1* gene mutation analysis in colorectal cancer samples from the Pakistani population. Next, molecular modeling and simulation studies were performed to gauge the conformational switches at a respective residual level and their significance in protein-protein interaction. Collectively, our results may extend our insight into the association of amino acid in the pathogenesis of colorectal cancer.

## Methods

### Ethical declaration

The study was approved by the Institutional Review Board (IRB) of Quaid-i-Azam University, Islamabad, Pakistan. Informed consent (written) was obtained from all those participating in the study.

### Patient and sample selection

200 colorectal tumors samples of both male and female patients with sporadic or familial colorectal tumors and normal tissues were taken randomly from Department of Urology, AFIP, Rawalpindi, Pakistan. At the time of biopsy patient age ranges were 32–78 years.

Clinical and demographic features were recorded, including age at the time of diagnosis, gender, family history, cell type, disease localization, stage and grade of a tumor (Table 1).

### DNA isolation

Before DNA extract, the presence of tumor tissue in FFPE blocks was examined and authenticated by an expert histo-pathologist. DNA was extracted from formalin-fixed and paraffin-embedded (FFPE) tissue specimens by using DNA extraction kit (Qiagen), following standard kit protocol [30].

### Mutation analysis of exon 3 of CTNNB1 gene

The extracted genomic DNA from tumor and normal control tissue (*n* = 200) was used as a template for amplification of exon 3 of the *CTNNB1* gene. The amplification was carried according to standard procedure in a total volume of 25 μl, containing 40 ng genomic DNA, 20 pmol of each primer, 200 μM of each deoxyribonucleotide, 1 unit of Taq DNA polymerase and 2.5 μl reaction buffer (MBI Fermentas, York, UK). The thermal cycling conditions used included 94 °C for 6 min, followed by 40 cycles of 94 °C for 45 s, 60 °C for 30 s, 70 °C for 30 s, and final extension at 70 °C for 8 min. Polymerase chain reaction (PCR) products were resolved on 2% agarose gel, stained with ethidium bromide and genotypes were assigned by visual inspection.

Primers were designed by software primer3 and used forward and reverse primers were.

*BC-F*: 5′-CCAATCTACTAATGCTAATACTG-3′ and.

*BC-R*: 5′ GCATTCTGACTTTCAGTAAGGC-3′, which yielded a product of 240 bp in size while amplification. Purified PCR Products were sequenced with forward and reverse primers by Macrogen Inc. (www.macrogen.com). BIOEDIT sequence alignment editor version 6.0.7 was used to identify the sequence variants. Also obtained sequences were investigated and aligned with CTNNB1 reference sequence, NG_013302.1 (www.ncbi.nlm.nih.gov).

### Immunohistochemistry

β-catenin protein expression was examined by immunohistochemistry which was carried on formalin fixed, paraffin embedded tissue using an anti β-catenin monoclonal antibody (Sigma-Aldrich) and executed with ultraView DAB Detection Kit (Ventana, Arizona, USA) on a BenchMark XT automated staining system (Ventana, Arizona, USA).

### 3D structure prediction

In the absence of a well-described or experimentally resoluted full length three dimensional protein structure, comparative modeling being the most precise computational approaches to make a consistent tertiary protein structure through sequence information [31]. Through homology modeling approach (http://www.rcsb.org), full length three dimensional structure of human β catenin protein was constructed using Swiss model (https://swissmodel.expasy.org/) and fold recognition method using MUSTER [32]. Next, the structure of mutated CTNNB1 (S33F and T44A) were predicted through the Swiss model (https://swissmodel.expasy.org/). Stereochemistry and validity of constructed wild type and mutated 3D structure of the human β-catenin protein was evaluated by Ramachandran polt [33] ProQ (Arjun et al., 2015) and Verify3D [34] and coarse packing quality evaluated with WHAT IF [35]. Auxiliary investigation of packing and stereochemistry was performed out and owing to several inconsistency; refinement was prepared to the preliminary model to clasp a superior model for further analysis. Energy minimization and structure refinements were made using GROMMACS available in Chimera 1.5.6 [36] and VEGA ZZ (http://www.ddl.unimi.it)*.*

### Molecular docking

AutoDOCK 4.0 was used to perform molecular docking of CTNNB1^wt^ with GSK3 and TrcPB1 [37]. Three-dimensional structure of GSK3 (PDB ID: 1GNG) and TrCP1 (PDB ID: 1P22) were retrieved through PDB (protein databank). The retrieved structure were subjected to geometry optimization using MMFF94 force field embedded in Chimera tool. The annealing parameters for hydrogen bonding and Van der Waals interactions were set to 4.0 A° and 2.5 A°, respectively. Grid map on the whole protein was generated with grid parameter of 80 _ 80 _ 80 points along with spacing of 0.875 A°. For each docking experiment the number of runs was set to 100. The Lamarckian genetic algorithm and empirical free energy function were applied using following parameters: a maximum number of 27,000 generations, population of 150 randomly placed individuals a crossover rate of 0.80 and the number of energy evaluations was 2.5 × 106 and mutation rate of 0.02, rest of the docking parameters were set to the default. Based on RMSD value of receptor ligand complex conformations cluetrs were generated. The best docked complex for CTNNB1 with GSK3 and TrcP1 were selected on the basis binding free energy value using ligplot [38], Discovery Studio visualizer *(*http://accelrys.com/products/collaborative-science/biovia-discovery-studio*)* and UCSF chimera [36] molecular interactions were mapped.

### Molecular dynamic simulations

To evaluate the stability, conformational changes and folding of CTNNB1 protein parallel molecular dynamic (MD) simulations experiments were performed with CTNNB1^WT^ and mutant S33F and T41A respectively. Using GROMACS 4.5 package [39], running on high performance OpenSuse linux system all MD simulations were performed.. All the systems were solvated using TIP4P water model in a periodic box [40],, followed by the addition of Na^+^ and Cl^ˉ^ counter ions to neutralize the systems. Under constant temperature (300 K) and pressure (1 atm) all MD simulations were run for 20 ns time scale To calculate electrostatic interactions PME (Particle Mesh Ewald) algorithm was used in all calculations. To analyze the stability and behavior of wild type and mutant system**,** VMD [41], PyMol (http://www.pymol.org) and GROMACS tools were used.

## Results

### DNA sequencing

**Fig. 1:**
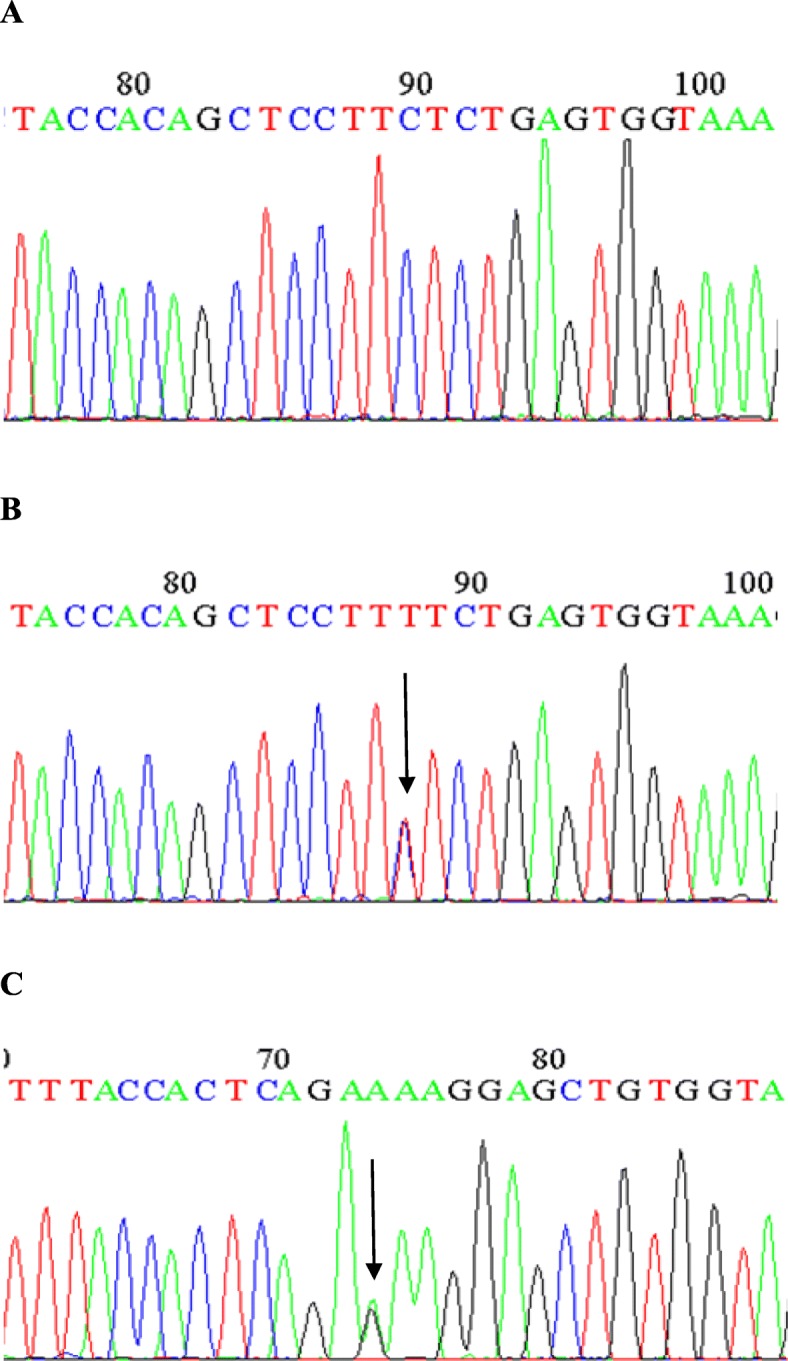
Sequence analysis of CTNNB1 exon 3 in colorectal cancer tissue and corresponding normal tissue of the same patient. Heterozygous mutation at codon 33 (TCT to TTT) in tumor. Chromatogram of *CTNNB1* exon 3, (**a**): DNA from normal tissue with wild type codon TCT (S33) sequenced with forward primer and (**b**), (**c**): DNA from tumor tissue with heterozygous mutation S33F (TTT > TCT) sequenced with forward primer and with reverse primer

**Fig. 2:**
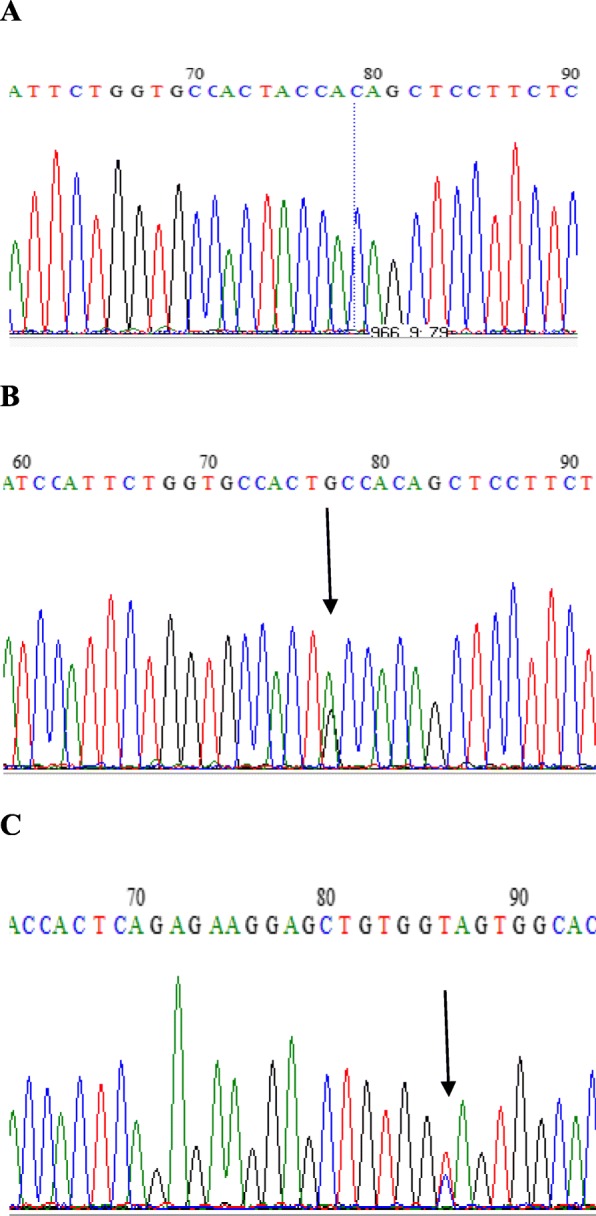
Sequence analysis of CTNNB1 exon 3 in colorectal cancer tissue and corresponding normal tissue of the same patient. Heterozygous mutation at codon 41 (ACC to GCC) in tumor. Chromatogram of *CTNNB1* exon 3, (**a**): DNA from normal tissue with wild type codon ACC (T41) sequenced with forward primer and (**b**) and (**c**): DNA from tumor tissue with heterozygous mutation (ACC > GCC) sequenced with forward primer

Sequence analysis revealed two activating mutations (S33F and T41A) in exon 3 of *CTNNB1* gene involving the transition of C.T and A.G at amino acid position 33 and 41 respectively (p.C33T and p.A41G). This substitution resulted in replacing a hydrophilic neutral serine to a hydrophobic phenylalanine at amino acid position 33 [TCT (Ser) → TTT (Phe)] (Fig. 1) and a polar threonine was converted to non-polar alanine at amino acid position 41 [ACC (Thr) → GCC (Ala)] (Fig. 2), of exon 3 of *CTNNB1* gene. Corresponding non-tumorous tissue did not reveal a mutation. From a 65 year old female and 54 year old male subject with cecal cancer and retroperitoneal mass, the tumor and adjacent normal tissue were acquired. Our observation revealed that β-catenin may have a vital part in the progression of colorectal carcinoma and that activating mutation of the β-catenin gene may substitute bi-allelic *APC* inactivation in this tumor type in Pakistani Population.

**Table 1:** Demographic and clinical data of colorectal cancer patients (*N* = 200)

Sex	Number of patients	Average age	localization	Cell type	Grade	Stage	Smoker (S) Non smokers (NS)	HRT therapy Yes/ no	Family history of CRC
Males (M)	136	53.67	Rectum 28 (M) 08 (F)	Adenocarcinoma 100 (M) 58 (F)	Well Differentiated 106 (M) 60 (F)	T0 NO MO T1 N1 Mx T2 NO Mx T2 N1 Mx T2 Nx Mx T3 NO MO T3 N0 M1 T3 N0 Mx T3 N1 Mx T3 N2 MO T3 N2 M1 T3N2Mx T4 N0 Mx T4 N1 M1 T4 N2 Mx T4 N3 M1	60 (S) 76 (NS)	NO	130 NO 6 yes
Females (F)	64	56.03	Colon 92 (M) 49 (F)	Mucinous Carcinoma 30 (M) 04 (F)	Moderately Differentiated 27 (M) 03 (F)		4 (S) 60 (NS)	22 yes 42 No	2 yes 62No
			Recto sigmoid 17 (M) 04 (F)	Signet Ring cell carcinoma 06 (M) 02 (F)	Poorly Differentiated 03 (M) 01 (F)				
			Cecum 09 (M) 03 (F)

**Fig. 3:**
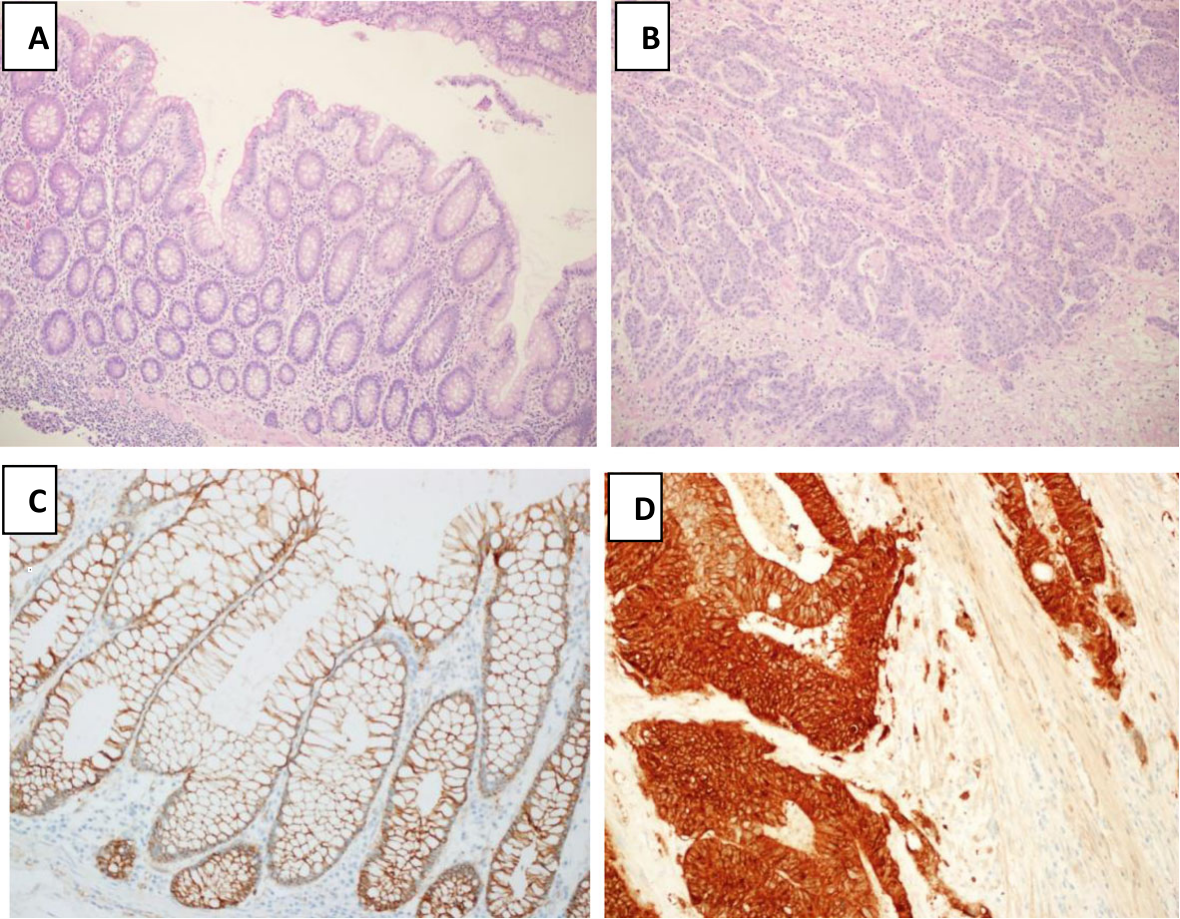
Protein expression of β-catenin in normal colon and colorectal cancer tissue from the same patient. Normal colon (**a**) and colorectal cancer tissue (**b**) stained with H & E. Slide showing strong nuclear and cytoplasmic localization of β-catenin in the tumor cells (**d**) and weak staining in normal tissue (**c**)

We carried out an immune-histochemical examination on formalin-fixed, paraffin-embedded tissue using anti β-catenin monoclonal antibody to explore the protein expression level of β-catenin in the tumor and the adjacent normal tissue. Increased protein expression was shown by tumor cells with the S33F and T41A mutation as compared to normal adjacent cells with wild type β-catenin protein (Fig. 3). The enhanced protein expression in S33F and T41A mutated tumor cells were localized to the cytoplasm as well as in nuclei.

### Mapping and characterization of mutations in crystal structure of β-catenin

**Fig. 4:**
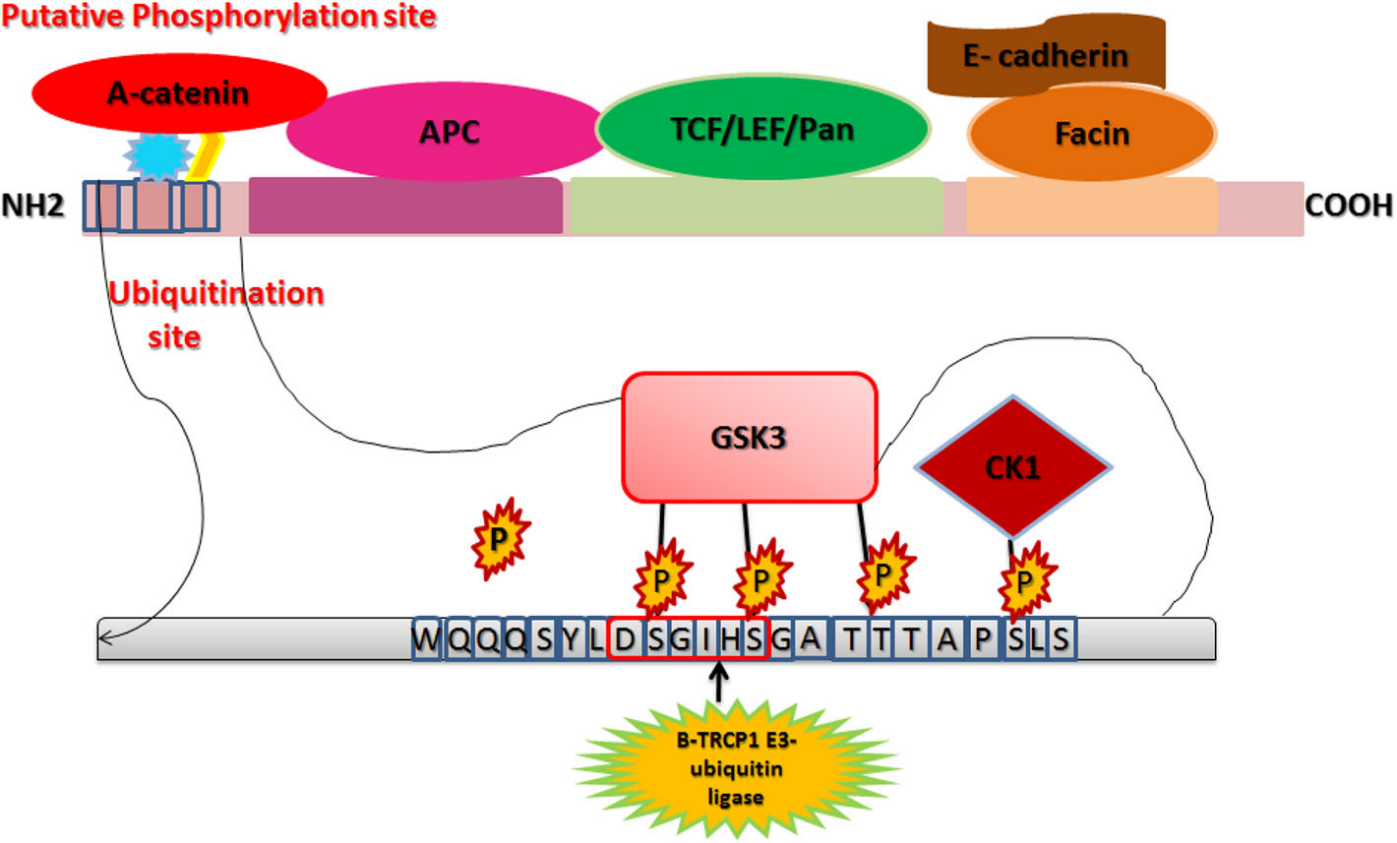
Regulation of CTNNB1 at molecular level

Due to lack of full length CTNNB1 structure, de novo loop modeling and protein threading techniques were used to model N-terminus region (1–148 amino acids). To characterize and assess the structural impact of mutations on CTNNB1 protein the reported mutation in the present study were mapped upstream of the armadillo repeat domain in the N-terminus intrinsically disordered region. The N-terminal disordered region of CTNNB1 holds a conserved short linear motif phosphorylated by GSK-3 and are accountable for binding of TrCP1 (also known as β-TrCP) E3 ubiquitin ligase with phosphorylated CTNNB1 [42, 43] (Fig. 4). Axin helps GSK-3-mediated phosphorylation of β-Catenin by bringing them in close proximity [44]. Ramachandran plot for the predicted CTNNB1^WT^ model revealed the occurrence of approximately 97.37% residues in the candidate region. Besides, other factors including peptide bond planarity, non-bonded interactions, Ca tetrahedral distortion, main chain H-bond energy, poor rotamers and overall G-factor for the modeled structures lied within the favorable range. The CTNNB1^WT^ structure was used to model mutated *CTNNB1*.

### Binding orientation and interaction mode analysis

**Fig. 5:**
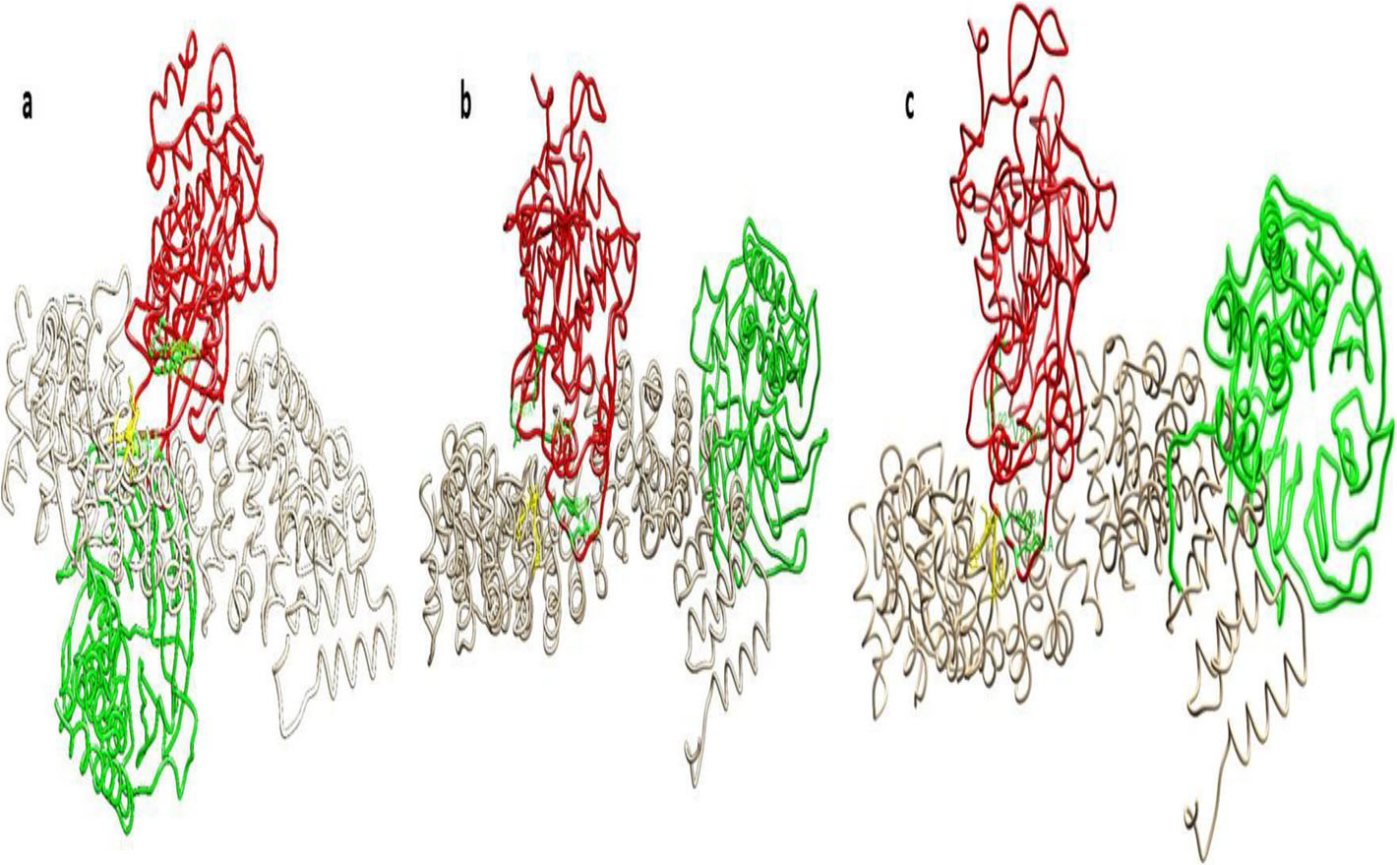
Molecular docking analysis of CTNNB1^WT^ and CTNNB1^MT (S33F and T41A)^ with GSK3 and TrCP1. **a** Wild type (**b**) CTNNB1^S33F^ and (**c**) CTNNB1^T41A^ Tan color ribbon represents CTNNB1 along with destruction motif shown in yellow. GSK3 is shown in red ribbon with interacting residues shown in green sticks. TrCP1 is represented in green ribbon

**Fig. 6:**
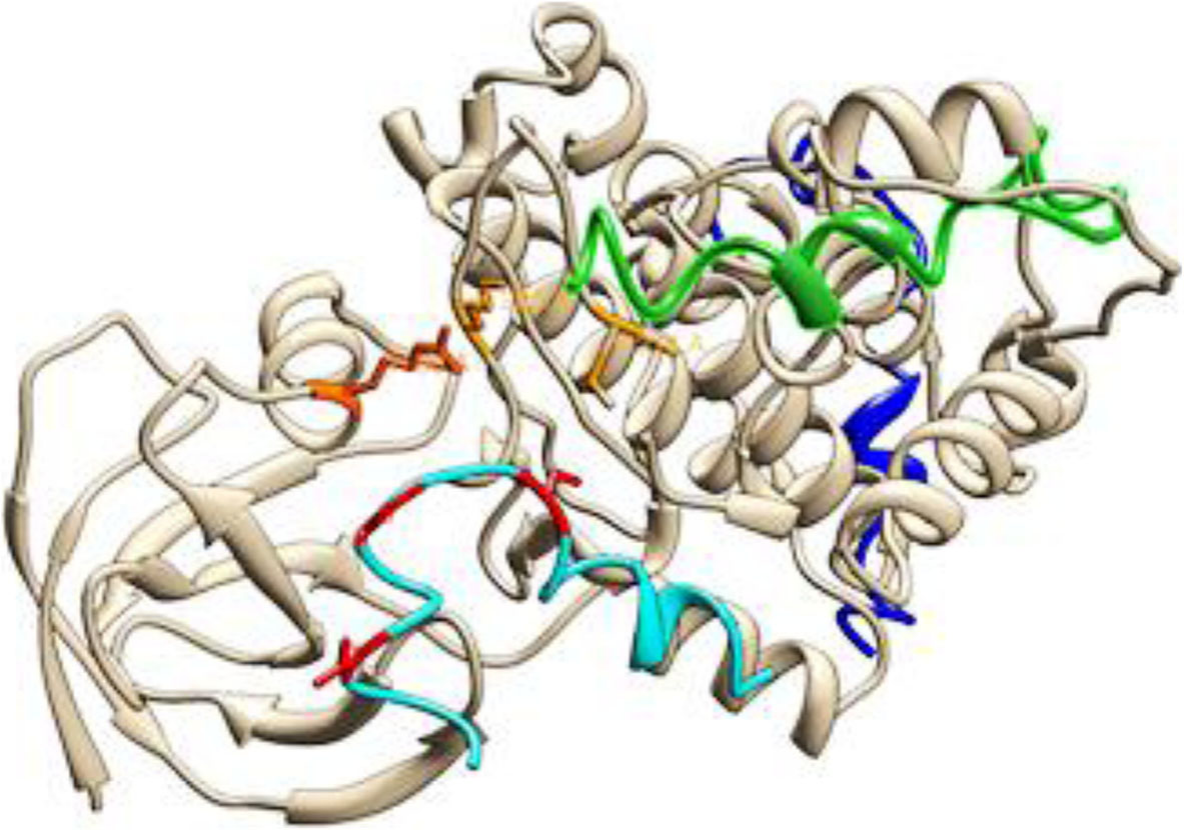
Molecular docking complex of CTNNB1^MT (S33F and T41A)^ with GSK3. Interaction of GSK3 and its substrate of CTNBB1 motif. The cyan color show the wild motif, green show mutant S33F and blue showed the mutant T41A. The red color sticks represents interacting residue of CTNBB1 and interacting residue of GSK3 are shown in orange sticks

To evaluate the conformational modifications in CTNNB1 upon interaction with GSK3 and TrCP E3 ubiquitin–ligase, 3D structure of mutant and wild type full length CTNNB1 was investigated. Docking analysis revealed most pronounced changes in the N-terminus disordered region of *CTNNB1*. GSK3 phosphorylates β-catenin at S33, S37 and T41 residues [43]. In-vivo studies revealed that mutation of CTNNB1 at S/T position abolish its phosphorylating by GSK3 because it is a processive kinase that sequentially phosphorylates S/T pentad repeats from the carboxy- to amino-terminal direction [31, 32, 45]. In the present study docking analyses of CTNNB1^WT^-GSK3 complex indicated that the S33, S37, and T41 residues of CTNNB1 involved in an interaction with R92, R96, R180, K205, V214, I217, Y288, and E290 of GSK3 (Fig. 5). A comparative docking analysis of an interaction of GSK3 with CTNNB1^WT^ and CTNNB1^MT(S33F and T41A)^ revealed that due to mutations in CTNNB1 binding of GSK3 was abolished and it moved away from N-terminus disordered region containing GSK3 phosphorylation motif (Fig. 6). Overall the total binding energy for CTNNB1^WT^-GSK3 interaction was − 42.789KJ/mol while the binding interaction energy for CTNNB1^MT(S33F and T41A)^-GSK3 interaction was − 28.66KJ/mol and − 25.89KJ/mol respectively.

**Fig. 7:**
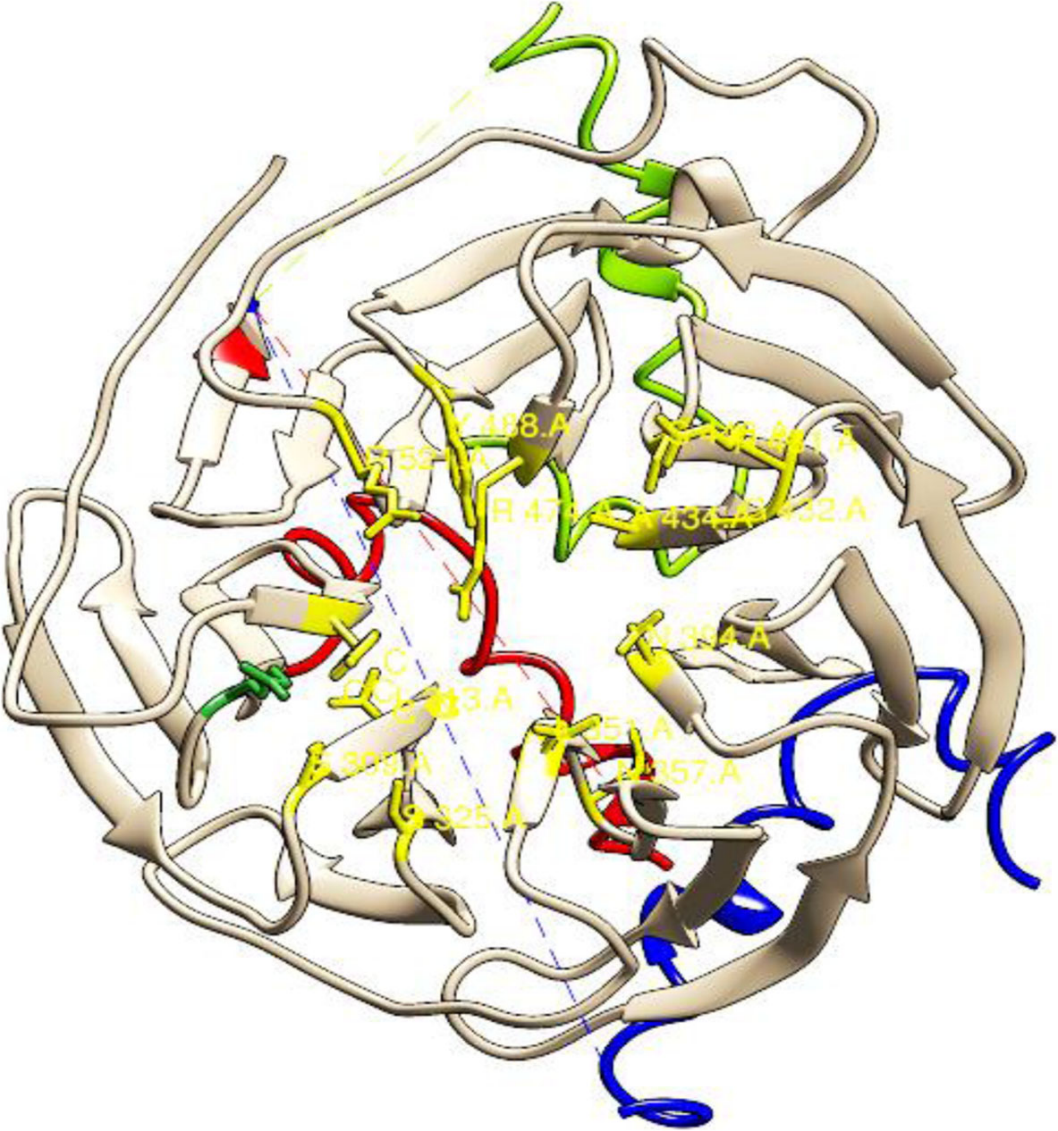
Molecular docking complex of CTNNB1 ^WT^ and CTNNB1^MT (S33F and T41A)^ with TrCP1. TrCp1 is shown in tan colour ribbon with interacting residues of narrow channel shown in yellow sticks. Wild type destruction motif of CTNNB1 is shown in red ribbon and loop form. S33F mutant CTNNB1 motif is shown in blue and T41A mutant is shown in green color

TrCP1 have two domain the N-terminal domain known as F box domain (residues 139 to 186), a C-terminal WD40-repeat domain (residues 253 to 545) and these two domain are linked by an -helical domain (residues 187 to 252) [33]. A WD40 domain structure has a narrow channel in the middle of the structure which has a narrow top face [34]. Docking analysis of CTNNB1^WT^-TrCP1 complex indicated that the destruction motif of CTNNB1 was buried at the top face of the TrCP1 narrow channel. Y271, S309, S325, R285, S448, G432, R474, Y488 of TrCP1 were involved in a number of binding interactions with N-terminus phosphorylated motif of CTNNB1^WT^. Comparatively, docking simulation of TrCP1 with CTNNB1^WT^ destruction motif and CTNNB1^MT(S33F and T41A)^ destruction motif revealed that due to mutation in phosphorylation site of destruction motif CTNNB1, its binding within the narrow channel of TrCP1 was obliterated it moved away channel of TrCP1 WD40 domain (Figs. 5 and 7). Overall the total binding energy for CTNNB1^WT^destruction motif-TrCP1 interaction was − 45.789KJ/mol while the binding interaction energy for CTNNB1 ^MT(S33F and T41A)^-destruction motif-TrCP1 interaction was − 20.66KJ/mol and − 15.89KJ/mol respectively. The docking analysis showed that substitution of S33F and T41A at the N-terminus phosphorylation motif of CTNNB1 eradicated its interaction with GSK3 and TrCP1.

### Molecular dynamics simulation analysis

**Fig. 8:**
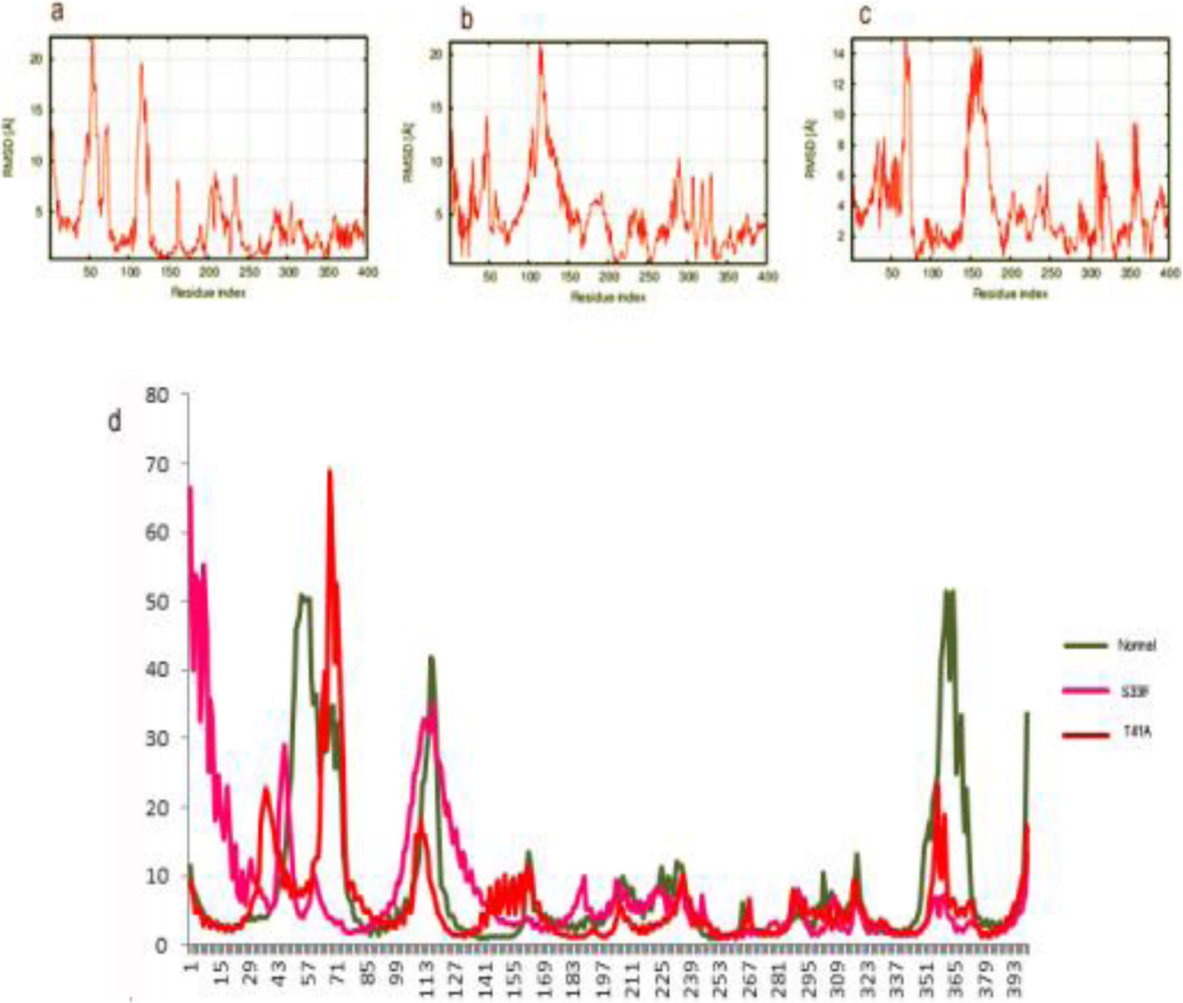
Plots to investigate the stability and fluctuation of MD trajectories for a wild type and mutant CTNNB1 systems. RMSD Plots computed through each system trajectory (**a**) Wild type CTNNB1 (**b**) S33F mutant of CTNNB1 (**c**) T41A mutant of CTNNB1. **d** RMSF plot for mutant and wild type CTNNB1 systems

The CTNNB1^WT^ and CTNNB1 ^MT(S33F and T41A)^ were further analyzed by molecular dynamics (MD) simulation assay in order to study the time-dependent behavior and to investigate the overall stability of the system. The stability of secondary structure elements and conformational changes of simulated complexes were assessed by plotting root mean square deviation (RMSD), root mean square fluctuation (RMSF), obtained throughout the trajectory. Our analysis indicated that the backbone RMSD score observed over a period of 20 ns remained stable (2 Å) throughout the simulation. These data validated the stability of CTNNB1^WT^ system during MD simulation. In general, N-terminal regions of exhibited more fluctuations than the C-terminal region, which is important for the GSK3 and β-TrCP binding. In CTNNB1^WT^ the high fluctuations observe between 1 and 110 residues and the systems remain stable at 200–400 residues. The RMSD analysis of CTNNB1^MT (S33F)^ indicated fluctuation in the N-terminus disordered region and extended up to 170 residues. Similarly, CTNNB1^MT (T41A)^ showed more fluctuations in the N-terminus disordered region and extended up to 200 residues (Fig. 8a-c). The high RMSD fluctuation rate of two mutant indicated that these mutant have a negative impact on protein stability.

Subsequent analysis of root mean square fluctuation (RMSF) per residue indicated a high fluctuation rate in the N-terminus regions upstream of armadillo repeat. However, armadillo fold were stable and exhibited minor fluctuations in CTNNB1^WT^ as compared to CTNNB1 ^MT(S33F and T41A)^ (Fig. 8d).

## Discussion

Colorectal cancer (CRC) is recognized to be the accumulative effect of numerous mutations within the cell that permit it to outflow growth regulation and regulatory mechanisms [46, 47]. Studies have proven that the accrual of gene mutations in clonal cell effects in the alteration from the normal colon epithelial cell into colorectal carcinoma [48]. In humans, *CTNNBI* gene mapped at 3p22 encodes the beta-catenin protein [48, 49]. This protein coordinates cell-cell adhesion and gene transcription [50]. Also performs as an intracellular signal transducer in the Wnt signaling pathway. The Wnt effector β-catenin is a transcriptional co-activator that can also mutate to a potent oncogene, while the canonical Wnt signaling pathway stabilizes β-catenin transcription [49, 50]. Both mutations and the overexpression of beta-catenin are allied with different cancers, including ovarian and endometrial carcinomas, hepatocellular carcinoma, colorectal carcinoma, malignant breast tumors, and lung cancer [51].

To our knowledge, this is the first comprehensive association study of *CTNNB1* gene with colorectal cancer patients in the Pakistani population. In this study, the incidence of mutations in the *CTNNB1* genes, as well as expression of the CTNNB1 protein in tumor tissue of 200 colorectal cancer subjects, were examined. The frequency of mutations in the *CTNNB1* gene, which codes for β-catenin, was rare, only two of 200 tumors analyzed were having a mutation in exon 3 at codon 33 and 41 in colorectal cancer tissues. This substitution resulted in replacing a hydrophilic neutral serine to hydrophobic phenylalanine at amino acid position 33 [TCT (Ser) → TTT (Phe)] and a polar threonine was converted to non-polar alanine at amino acid position 41 [ACC (Thr) → GCC (Ala)] of exon 3 of *CTNNB1* gene. Corresponding non-tumorous tissue did not reveal a mutation. Our finding comprehends the previous study carried out by Alomar and colleagues which screened *CTNNB1* gene in 60 colorectal cancer patients from Kingdom of Saudi Arabia (KSA) and revealed an activating mutation (S33F) in one of the tumor samples [52]. Our observations also analyzed the grade of β-catenin protein of the *CTNNB1* gene in colorectal cancer in Pakistani population. Strong increases of cytoplasmic and nuclear β-catenin concentration in the malicious cells of two of the 200 examined cases were seen when compared with normal adjacent tissue. Our results proposed a conceivable role of β-catenin accumulation due to the S33F and T41A mutations in the pathogenesis of colorectal cancer in two of the patients involved in our study. Similar observation was reported by Michiko and colleagues revealing the nuclear accumulation of β-catenin in colorectal cancer [53].

*CTNNB1* is phosphorylated by GSK3 involves a priming kinase that performs on a four serine (S)/threonine (T) (S33, S37, T41, and S45) amino acid C-terminal to a GSK3 phosphorylation site. Phosphorylated amino acids of the priming site bind to the catalytic pocket in GSK3, created by the amino acids R96, R180, and R205, and allow further phosphorylation through GSK3 [54]. These phospho-S/T residues are critical for β -catenin detection by the F box protein β-Trcp, which is the important player of ubiquitination device [55–59]. The importance of S33 S37, T41, and S45 phosphorylation in β-catenin degradation is emphasized by the surveillance that mutations at these S/T residues recurrently arise in human colorectal cancer and numerous other malignancies, which are allied with and most likely occurred by the decontrolled accumulation of β–catenin [26, 27, 60, 61]. Through our in silico deep structural analysis, we mapped docking sites of GSK3 and TrCP1 with N-terminus of CTNNB1 which clearly revealed interaction of GSK3 and TrCP1 and putative phosphorylation motif of CTNNB1. We are tempting to speculate that our findings open a room for cancer researcher through a functional interplay between CTNNB1, GSK3, and TrCP1. Molecular docking and dynamic simulation analysis of an interaction of GSK3 with CTNNB1^WT^ and CTNNB1^MT(S33F and T41A)^ revealed that due to mutation in CTNNB1 binding of GSK3 was eliminated. Comparatively, docking simulation of TrCP1 with CTNNB1^WT^ destruction motif and CTNNB1^MT(S33F and T41A)^ destruction motif revealed that due to a mutation in the phosphorylation site of destruction motif CTNNB1, its binding within the narrow channel of TrCP1 was abolished. The intertwined relationship of CTNNB1, GSK3, and TrCP1 could be a novel and interesting area for cancer therapeutic development.

## Conclusion

In summary, the present study on Pakistani population revealed the association of two non-synonymous polymorphisms in the *CTNNB1* gene with colorectal cancer. These genetic variants led to the accumulation of *CTNNB1*, a hallmark of tumor development. Further molecular modeling, docking, and simulation approach revealed significant conformational changes in the N-terminus region of normal to mutant *CTNNB1* gene critical for binding with GSK3 and TrCp1. Analysis of structure to functional alterations in the *CTNNB1* gene is crucial in understanding downstream biological events. Our observations need to be deep-rooted on a larger number of tumors in Pakistani population and we suggest that the identification of β-catenin/ *CTNNB1*genes expressed in colon cancer cells may offer novel opportunities for developing therapeutics against targets with a critical role in colon cancer.

## Data Availability

The datasets used and/or analysed during the current study available from the corresponding author on reasonable request.

## Abbreviations

APC: Adenomatous polyposis coli
CRC: Colorectal cancer
CTNNB1: Beta catenin
DAB: 3, 3‟-diaminobenzidine
*EMT*: Epithelial to mesenchymal transition
FFPE: Formalin-fixed and paraffin-embedded
GSK-3β: Glycogen synthase kinase 3-B
MD: Molecular dynamic
MUSTER: MUlti-Source ThreadER
PME: Particle Mesh Ewald
RMSD: Root mean square deviation
RMSF: Root mean square fluctuation
TCF/LEF: T-cell factor/lymphoid enhancer-binding factor
TP: Tumor protein
TrCp1: Transducin repeat containing E3 ubiquitin protein ligase
