# Correction to: Screening and computational analysis of colorectal associated non-synonymous polymorphism in CTNNB1 gene in Pakistani population

**DOI:** 10.1186/s12881-020-0979-4

**Published:** 2020-02-24

**Authors:** S. Razak, N. Bibi, J. A. Dar, T. Afsar, A. Almajwal, Z. Parveen, S. Jahan

**Affiliations:** 1 Department of Community Health Sciences, College of Applied Medical Sciences, King Saud University, Riyadh, Saudi Arabia; 2 Reproductive physiology lab, Department of Animal Sciences Quaid-i-Azam University, Islamabad, Pakistan; 3 Department of Bioinformatics, Shaheed Benazir Bhutto Women University, Peshawar, Khyber Pakhtunkhwa Pakistan; 4 Department of Zoology, College of Science, King Saud University, Riyadh, Saudi Arabia; 5 Faculty of Biological Sciences Quaid-I-Azam University Islamabad and University of Iceland, 101 Reykjavik, Iceland

**Correction to: BMC Medical Genetics (2019) 20:171**

**https://doi.org/10.1186/s12881-019-0911-y**

Following publication of the original article [1], the authors have flagged that the article has published with an error in the order of the affiliations.

Namely, in the affiliations list (under the ‘Author details’) affiliations ‘1’ and ‘2’ are interchanged; affiliation ‘1’ should be ‘2’, and vice-versa.

Please find the corrected order of affiliations in this correction.
